# One Season in Professional Cycling Is Enough to Negatively Affect Bone Health

**DOI:** 10.3390/nu15163632

**Published:** 2023-08-18

**Authors:** Francisco Javier Martínez-Noguera, Pedro E. Alcaraz, Raquel Ortolano-Ríos, Cristian Marín-Pagán

**Affiliations:** 1 Research Center for High-Performance Sport, University of Murcia, Campus de los Jerónimos, Guadalupe, 30107 Murcia, Spain; palcaraz@ucam.edu (P.E.A.); cmarin@ucam.edu (C.M.-P.); 2 Sports Physiology Department, Faculty of Health Sciences, UCAM Universidad Católica San Antonio de Murcia, 30107 Murcia, Spain; rakortrios@gmail.com

**Keywords:** endurance, bone mineral density, T-score, osteoporosis and osteopenia

## Abstract

Cycling is a very popular sport worldwide, and several studies have already indicated that cycling at various levels has a negative impact on bone health. This is of concern to both performance and health managers of many cycling teams at different levels because of its economic and social impact. Based on the scientific literature, we hypothesize that a single season at the professional level can negatively affect bone health status. The aim of this study was to assess how professional cycling affects bone health markers after one season. Densitometry was used to measure the bone mineral density (BMD), bone mineral content (BMC), bone area (BA), fat mass (FM), fat-free mass (FFM), T-score and Z-score in professional cyclists after one season. After one season at the professional level, cyclists’ BMD decreased significantly in the legs, trunk, ribs and pelvis (*p* ≤ 0.05). BMC decreased in the arms and spine (*p* ≤ 0.05). BA decreased significantly in the arms and spine (*p* ≤ 0.05). In addition, a significant decrease in Z-score (*p* ≤ 0.05) and a decreasing trend in T-score and total BMD (*p* = 0.06) were observed. One season of professional cycling is enough to negatively affect bone health status.

## 1. Introduction

In recent years, there has been a growing concern within sports science regarding bone health, especially in non-impact sports [1,2,3,4,5,6,7]. In particular, studies that have examined cyclists have demonstrated low values in bone health markers, which may consequently increase the risk of injury or early onset of bone disease (osteoporosis) [1,2].

This problem is multifactorial for cyclists. One of the factors is the lack of force impact that would stimulate bone remodeling by nature of the sport [8]. Another factor is energy availability (EA), specifically the amount of dietary energy available after exercise training for all metabolic processes expressed in relation to fat-free mass (FFM) [9]. For example, athletes who train and compete in endurance races (i.e., long distance) are more likely to have energy deficiencies [9]. This is due to the fact that cyclists use carbohydrate- or energy-restrictive diets to reduce body mass and fat mass with the aim of improving their power/weight ratio, as this is one of the best indicators of performance, especially in competitions with a lot of uphill terrain [10]. Since a lighter cyclist expends less energy than a heavier rider to maintain the same speed on uphill terrain, their fatigue on long climbs will be delayed. These energy imbalances are related to reproductive function and bone health in female and male athletes, also known as the “Relative Energy Deficiency in Sport” (RED-S) [11]. Energy deficit-related suppression of hormones, such as testosterone, triiodothyronine (T3) and leptin, suggests the dysregulation of the reproductive system and energy metabolism in male athletes [12,13].

Increased calcium loss through sweat in elite cyclists (up to 150 mg/h) could be another factor that negatively affects bone mineral density (BMD) [14]. As a consequence, there is an increase in parathyroid hormone concentrations that would promote bone demineralization to increase serum calcium levels [15]. If this mechanism is chronically activated, it may be a contributing factor to the low BMD of elite cyclists [16], although calcium loss through sweat is currently being questioned [17].

Bone health problems in cycling develop over a prolonged period with several influencing factors, where poor nutrition and lack of loading due to the mode of exercise are key factors [11,18,19]. It is known that low long-term energy availability is related to poor bone health in both male and female athletes [11,18,19,20], and road cycling does not produce significant osteogenic benefits compared to weight-bearing sports [21]. Cycling improves the cardiovascular system with decreased cardiovascular risk factors as well as reduced cancer risk [22]. However, as a non-weight-bearing sport, cycling is often associated with lower levels of bone mass [23], with two thirds of adult professional and masters road cyclists classified as osteopenic [24]. This situation can therefore lead to the development of osteoporosis, which typically affects older populations and is characterized by a deterioration of the microarchitecture of bone tissue and BMD, leading to increased bone fragility and susceptibility to fractures [25]. In addition, it has been shown that the level of practice and/or years of training are factors that could increase the risk of low bone mass [21].

There are many studies that have evaluated the effect of years of training and competition of high-level road cyclists (well-trained, amateur and professional cyclists) on markers of bone health [1,2,26]. Recently Martínez-Noguera et al. [2] found lower levels of BMD, bone mineral content (BMC) and bone area (BA) in professional cyclists compared to amateurs. A few years before, Klomsten et al. [1] observed that Norwegian national elite road cyclists had lower BMD compared to runners, and a large proportion were classified as having low BMD (Z-score ≤ −1), despite having performed heavy endurance training. Previously, Piotrowska et al. [26] found no significant change in BMD in any age or sex group in amateur cyclists during one season.

To date, we have not found any studies on professional road cyclists that assessed changes in bone health markers longitudinally. Therefore, the main objective of this study was to evaluate changes in bone health markers in professional road cyclists over two seasons. We hypothesized that there would be a negative effect on BMD and BMC in professional cyclists after two competitive seasons.

## 2. Methodology

### 2.1. Subjects of Study

Eighteen male professional cyclists completed this study (Table 1). The professional cyclists (ethnicity: 14 Caucasian and 4 Hispanic) competed in Union Cycliste Internationale (UCI) PRO TOUR competitions and participated in major UCI stage races (Vuelta a España, Giro d’Italia and Tour de France). The PRO cyclists were recruited on the basis of the following criteria: (i) aged between 20 and 40 years, (ii) enrolled in a registered professional team and (iii) have raced in at least one of the main 3-week-stage races in recent years. Additionally, exclusion criteria included riders who could have had or had rickets, metabolic diseases suffered in infancy and thyroid diseases.

The subjects signed an informed consent form prior to enrollment. The study was performed under the guidelines of the Declaration of Helsinki Declaration for Human Research [27] and was approved by the Ethics Committee (CE091802).

### 2.2. Study Protocol

This study has a longitudinal experimental design that required each cyclist to visit the laboratory in the 2018 and 2019 pre-season. Subjects were measured from 8:30 to 11:00 a.m. on an empty stomach (no food and liquid intake). Prior to data collection, subjects were advised of the nature and possible risks of the project, and informed written consent was obtained.

### 2.3. Dual-Energy X-ray Absorptiometry (DXA)

Body composition was assessed via whole-body DXA (XR-46; Norland Corp., Fort Atkinson, WI, USA). BMD (g/cm^2^), bone mineral content (BMC) (g), bone area (BA) (cm^2^), fat mass (FM) and fat-free mass (FFM) (g) were evaluated during morning fasting. In the preceding 5 years, no calibration errors were found, and there were no firmware or software updates. The measuring device had been checked by the manufacturer. All patients wore underwear without metal accessories during the assessments. The explorations and analyses were carried out by an experimented and certified technician. The repeatability, expressed as the coefficient of variation of BMD measurements, was 0.86% for the whole body. The anatomical segments of arms, legs, trunk, ribs, pelvis and spine were analyzed using the whole-body scanner.

### 2.4. Statistical Analyses

Statistical analysis was carried out with SPSS 21.0 software (International Business Machines, Chicago, IL, USA). Data are presented as mean ± standard deviation (SD). The Levene and Shapiro–Wilk tests were run to test for homogeneity and normality of the data, respectively. A paired-samples *t*-test was applied to compare BMD, BMC, BA, FT, FFT, T-score and Z-score between 2018 and 2019. Moreover, Wilcoxon signed-rank test was used when the data did not have a normal distribution. The level of significance was set at *p* ≤ 0.05. Furthermore, standardized mean differences were calculated utilizing Cohen’s effect size (ES) with a 95% confidence interval (CI) for all comparisons. Threshold values for the ES statistics were as follows: >0.2 small, >0.5 moderate and >0.8 large [28].

## 3. Results

**Figure 1:**
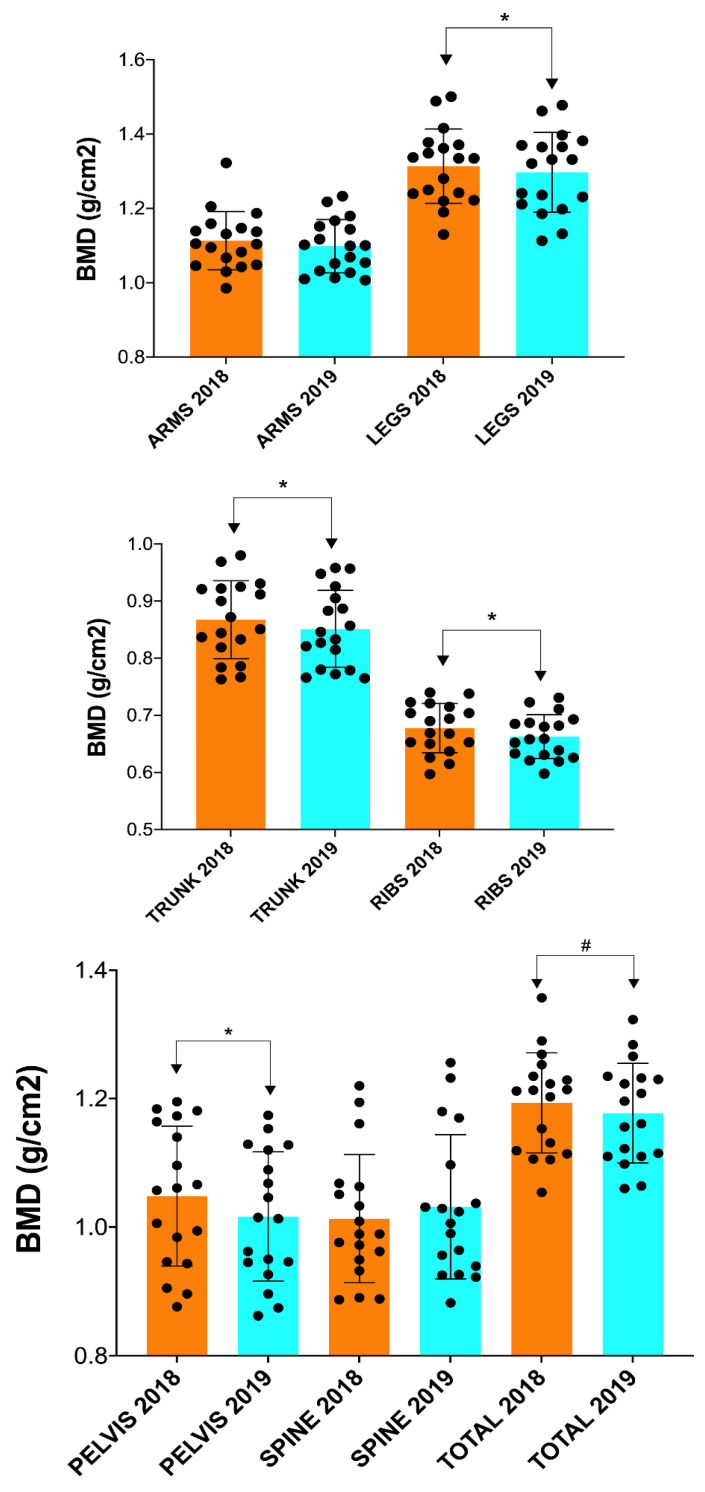
Changes in BMD after one season in professional cyclists in arms, legs, trunk, ribs, pelvis, spine and total. BMD = bone mineral density. * ≤ 0.05; # = in the range of 0.051–0.07.

Table 2 presents the comparison of BMD, BMC, BA, FM and FFM at the pre-season professional cycling in 2018 and 2019. After one year of training and competition at the professional level, a significant decrease in BMD was observed in the legs, trunk, ribs and pelvis (*p* ≤ 0.05), and a decreasing trend was observed in total BMD in professional cyclists (*p* = 0.061; Table 2 and Figure 1).

**Figure 2:**
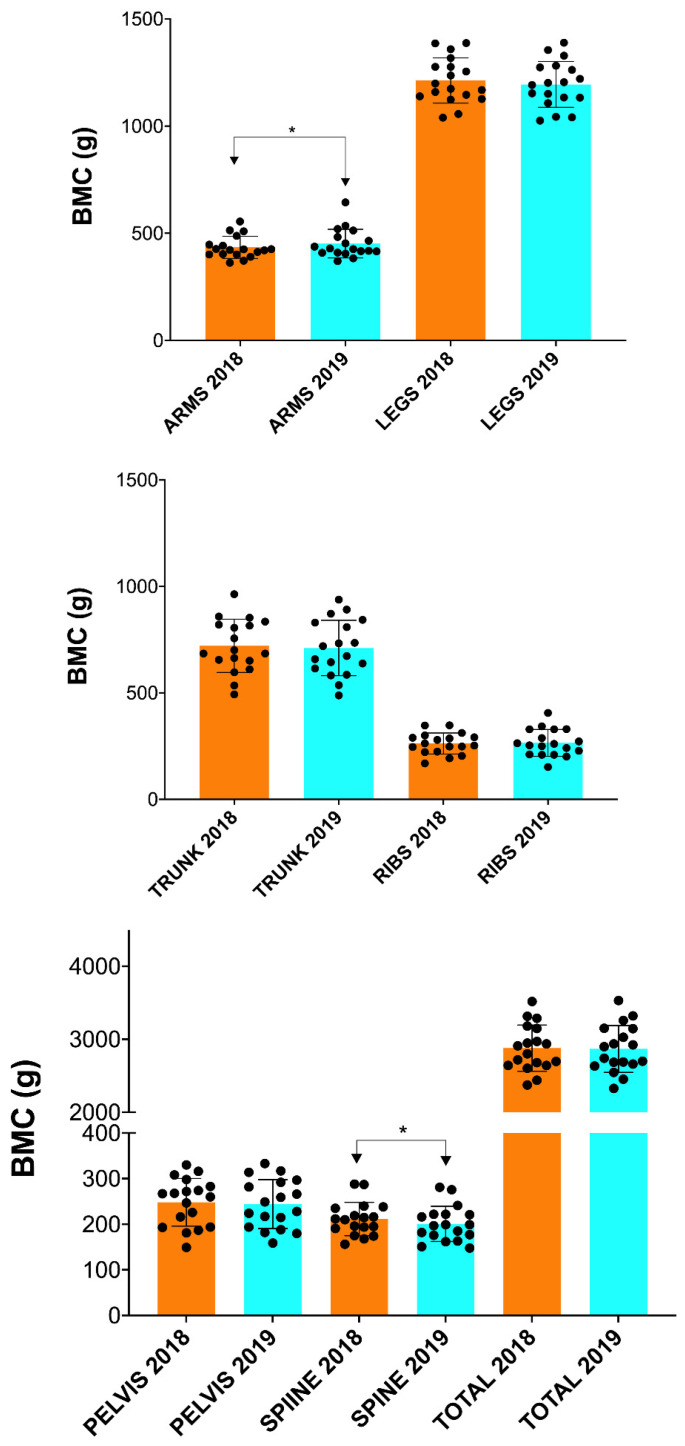
Changes in BMC after one season in professional cyclists in arms, legs, trunk, ribs, pelvis, spine and total. BMC = bone mineral content. * ≤ 0.05.

**Figure 3:**
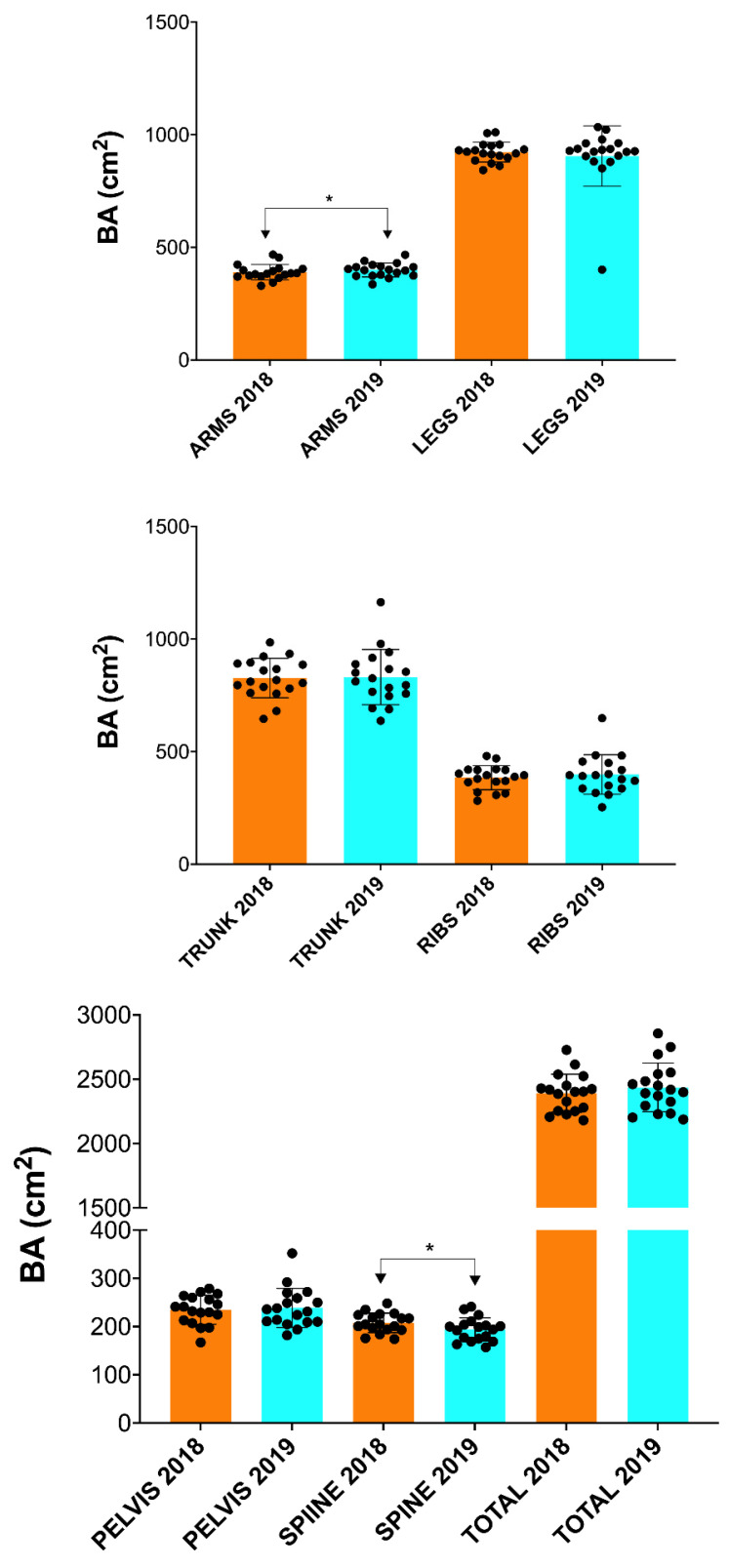
Changes in BA after one season in professional cyclists in arms, legs, trunk, ribs, pelvis and total. * ≤ 0.05.

**Figure 4:**
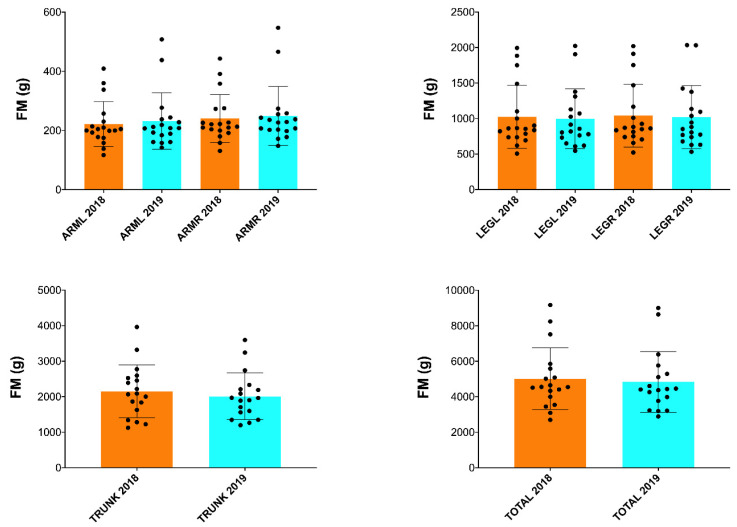
Changes in FM after one season in professional cyclists in arms, legs on the right and left side, trunk and total. FM = fat mass; FFM = fat-free mass; R = right; L = left.

**Figure 5:**
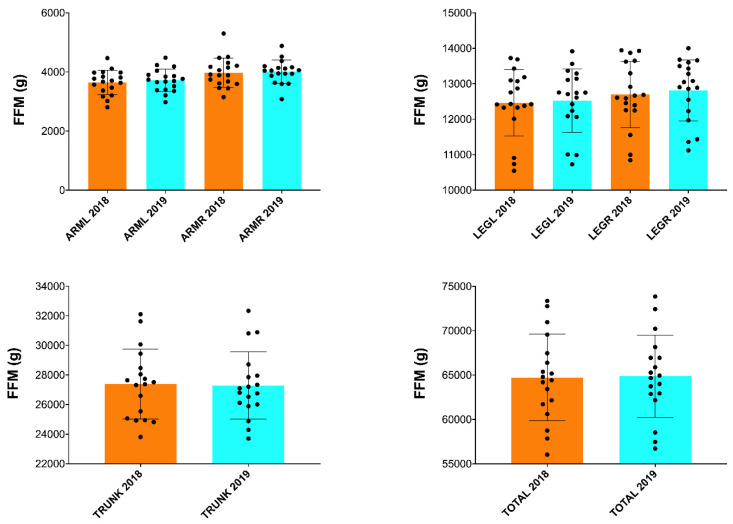
Changes in FFM after one season in professional cyclists in arms, legs on the right and left side, trunk and total. FM = fat mass; FFM = fat-free mass; R = right; L = left.

In addition, a significant increase in BMC (*p* ≤ 0.05; Figure 2) and BA (*p* ≤ 0.05; Figure 3) was found in the arms and spine after one season (Table 2). However, no significant changes in FM and FFM were observed in any of the anatomical areas analyzed (Table 2 and Figure 4 and Figure 5).

**Figure 6:**
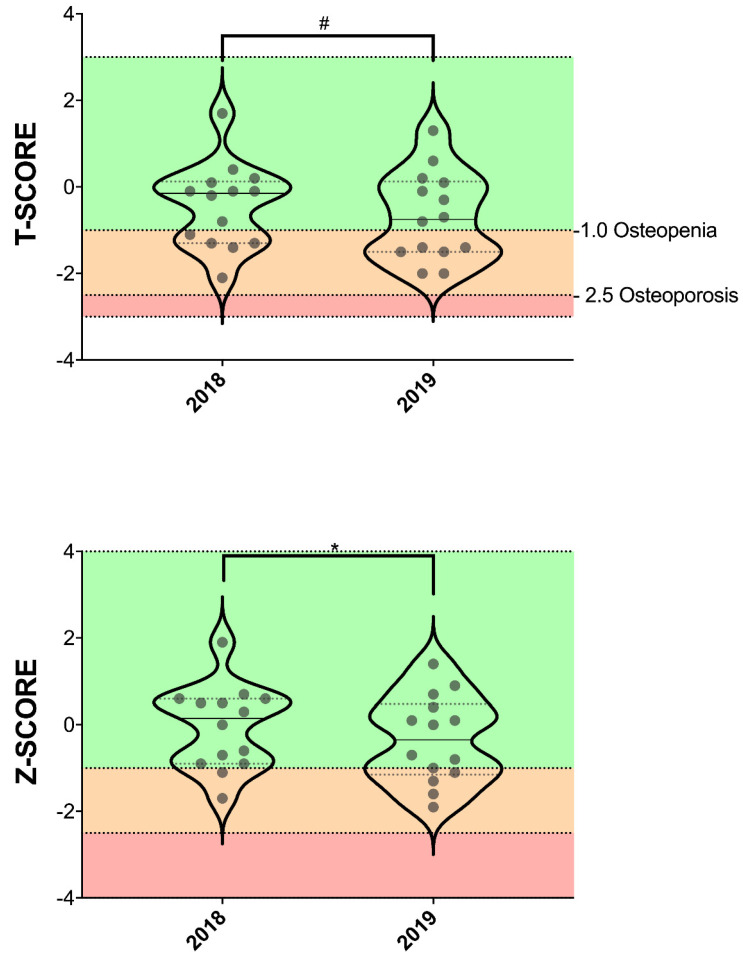
Changes in whole-body T-score and Z-score after one season in professional cyclists. * = ≤0.05; # = in the range of 0.051–0.07.

Moreover, professional cyclists showed a decrease in Z-score (−502%; *p* = 0.021; ES = 0.556) and a downward trend in T-score (−55.7%; *p* = 0.061; ES = 0.462) after one season of training and competitions (from 2018 to 2019; Table 2 and Figure 6). It should be noted that 5 of the 29 cyclists had osteopenia in 2018, and this figure increased to 6 in 2019.

## 4. Discussion

The main objective of this study was to evaluate the effect of a professional cycling season on markers of bone health and body composition. The main findings were a decrease in BMD, BMC and BA at the arm and spine level after one season of training and competition in professional cyclists. These results suggest that one season is sufficient to negatively affect markers of bone status in professional cyclists.

Specifically, we found a significant decrease in BMD in the legs, trunk, ribs and pelvis, with a downward trend in total BMD. To date, we have not found a study that has evaluated the effects of a cycling season on markers of bone status. However, in a recent study, we observed that professional cyclists had lower BMD compared to amateur cyclists [2]. Furthermore, all professional cyclists and 7 out of 15 amateur cyclists had a BMD below 1.033 g/cm^2^, which is the normal BMD cutoff value for men that was established by the North American Health Survey (NHANES III) [2]. Previously, Gonzalez-Aguero et al. [29] also demonstrated that young cyclists had lower BMD and BMC values compared to controls at the level of the radius and tibia. This implies that there is a negative effect on bone health from an early age in cyclists. Therefore, if this negative effect at the bone level appears in young cyclists and is prolonged throughout the competitive period, especially at the professional level, the risk of having a bone pathology (osteopenia or osteoporosis) would be higher. It is important to consider that in osteopenia, the BMD is between −1.0 and −2.5 SD compared to the BMD of healthy young people at peak bone mass, and osteoporosis occurs when BMD is ≥2.5 SD compared to the BMD of healthy young people at peak bone mass [30]. This implies that there is an increased risk of bone fracture in professional cyclists.

Another important bone health marker is BMC [24]. We found a significant decrease in BMC, as well as BA, in the arms and spine in professional cyclists after one season. Medelli et al. [24] reported similar values of BMC in professional cyclists compared to our cohort (2.8 kg vs. 2.9 kg, respectively). Furthermore, they found positive correlations between total BMC and weight (r = 0.760; *p* ≤ 0.001) and between total BMC and fat mass (R^2^ = 0.60; *p* ≤ 0.0001). These results are consistent with the positive correlation between BMC and FFM in professional and amateur cyclists (r ≤ 0.738; R^2^ ≤ 0.545; *p* ≤ 0.03) found by Martínez-Noguera [2]. Based on these findings, weight, fat mass and fat-free mass have an important relationship with BMC in cyclists. Moreover, according to our findings, we can establish that a professional cycling season can affect the BMC, especially at the levels of the arms and spine. It has been suggested that lean (muscle) mass may be a factor in determining bone mineral density by generating increased mechanical loading on bones, particularly weight-bearing bones [31]. On the other hand, the loss of bone mass due to both microgravity weightlessness and bed rest support this association [32]. The mechanism of the effect of weight on BMD is currently not described. But it has been hypothesized that it could be due simply to increased mechanical loading, or weight could be a surrogate marker for muscle strength, or body fat and nutritional, metabolic or hormonal status [33].

The WHO classifications for the diagnosis of osteopenia is a T-score value of >1.0 to <2.5 SD, whereas for osteoporosis the T-score cutoff is ≥2.5 SD below the young adult mean [34,35]. We observed a decrease in the Z-score and a decreasing trend in the T-score in professional cyclists after one season. Very few studies have used the T-score to assess bone health status in cyclists, primarily measuring BMD and BMC. Nichols et al. [36] observed that 67% of the master cyclists would be classified as either osteopenic (52%) or osteoporotic (15%) at either the spine and hip or both. Ten non-athletes (42%) would be classified as osteopenic, while none was osteoporotic. In addition, four (25%) young adult cyclists would be classified as osteopenic in the lumbar spine. However, Smathers et al. [37] observed no significant differences in T-score, Z-score and total body BMD between male competitive cyclists and untrained controls but found significantly lower values for T-scores and Z-scores for all of the spine sites in cyclists compared to controls. Based on these studies, we can affirm that cycling is a sport that can produce a decrease in bone health markers, such as T-score and Z-score, as well as BMD and BMC, compared to a cohort of untrained or non-athletes, increasing the risk of a bone fracture. We can also add that a single year of training and competition in professional cyclists is enough to negatively affect bone health status. Therefore, it is necessary to carry out an intervention that can prevent this deterioration of bone health in professional cyclists, but also at lower levels of practice.

The deterioration of bone health in cyclists is a multifactorial problem. Features such as low mechanical load on the skeleton, an energy deficit combined with high volumes of training and competition (500–1000 km/wk) or increased calcium loss through sweat with possible modulation of parathormone levels could contribute to this issue [15].

In relation to mechanical loading, it is well known that during exercise, the skeleton is exposed to different types of stresses (tissue deformation), generated by compression, tensile and torsional forces and shear force, and these stresses can occur at the same time and in the same bone [38]. Furthermore, running has been shown to produce tibial stresses 2–3 times greater than walking [38], and those relating to walking have been shown to be greater than cycling [39]. Thus, cycling as a sport is a poor bone simulator. Along this line, Scofield and Hecht [3] found that adolescent runners had higher levels of total BMD compared to cyclists and swimmers. It is likely that the optimal exercise to induce osteogenesis and bone anabolism (bone formation) is likely to differ by age, sex and individual [40], and even skeletal site, suggesting that only an individualized approach would provide accurate information to design the optimal osteogenic exercise regime [41].

Other possible factors responsible for low BMD and BMC levels in cyclists are low energy availability (LEA) and low body mass, which are implicated in compromised bone health in elite cyclists [18,42]. A high risk of LEA has been identified in both male and female elite cyclists (ref). In fact, bone strength can be influenced by LEA both directly and indirectly. The direct mechanisms are through suppression of triiodothyronine (T3), insulin-like growth factor 1 (IGF-1) and insulin, resulting in decreased bone formation. The indirect mechanism is through the reduction in reproductive hormones leading to increased bone resorption [43]. Loucks et al. [44] have shown that 45 kcal·kg^−1^ of fat-free mass (FFM)·d^−1^ is the amount of energy availability required to achieve an energy balance in young women. Energy availability levels below 30 kcal·kg^−1^ (FFM)·d^−1^ result in the suppression of luteinizing hormone (LH) pulsatility in women [45]. The decrease in LH together with other female hormones such as follicle-stimulating hormone, luteinizing hormone and estradiol, may be due to a functional hypothalamic amenorrhea (FHA) secondary to underweight, excessive exercise and/or high levels of stress, which commonly occurs in young female athletes [46]. Moreover, there may be a genetic factor that puts some female athletes more at risk for FHA [47]. Male athletes can also suffer from hormonal dysfunction, since, as some studies have shown, total and bioavailable testosterone levels are substantially reduced in certain men who exercise, particularly in endurance-trained athletes [48,49,50]. In this line, Wheeler and Hackney also showed that hormonal alterations in men were similar to those in women who developed exercise-related menstrual dysfunction [51,52]. Subsequently, Hackney et al. established the term hypogonadal male exercise dysfunction (EHMC) to describe this condition in men [53]. For example, basal testosterone levels well below the normal physiological range have been observed in elite athletes performing ultra-endurance events (races > 100 km, under extreme environmental conditions) [54,55].

On the other hand, it has been observed that competitive cyclists with LEA also had lower testosterone levels compared to cyclists who had adequate energy availability [56]. Therefore, the presence of both sexes in cyclists can produce an alteration of the hormonal axis, which as a consequence leads to a negative effect at the bone level. A recent paper by Fensham et al. [57] demonstrated that short-term carbohydrate restriction results in a decrease in markers of bone formation at rest and during exercise, with an additional exercise-related increase in a marker of bone resorption in race walkers. Furthermore, they showed that markers of bone formation during exercise are maintained with LEA even though resorption increased. Similar findings were found by Lombardi et al. [58] as they observed that a professional cycling competition (Giro d’Italia, 3 weeks long) increased undercarboxylated osteocalcin in the morning (before the competition) between days 1 and 12, indicating an increase in metabolic stress and bone resorption. Another hormone that may influence bone metabolism is cortisol, which has been shown to increase after completion of an ultramarathon (endurance sport) and correlates negatively with markers of bone formation [59]. In addition, it has recently been reported that serum cortisol concentration increased steadily after exercise relative to pre-exercise after 4 days of cycling training (3 h daily) [60]. The intense physiological and environmental stress of ultra-endurance events can induce acute reductions in testosterone and LH levels after the event, as well as increases in cortisol, suggesting a significant suppression of hypothalamic–pituitary axis functions [54,55].

At the metabolic level, an increase in sweat Ca^+^ loss during 2 h of moderate cycling has also been observed to be associated with a decrease in serum Ca^+^ accompanied by an increase in plasma parathyroid hormone concentration, which may promote bone resorption [61]. However, this parathyroid hormone response to exercise was attenuated when calcium supplementation was taken immediately before or during cycling [14]. Prolonged exercise has been shown to stimulate PTH secretion, but the effects of these transient increases in PTH on bone metabolism in the medium and long term are unknown [61].

Due to the findings found in our study and those presented in the discussion, it is evident that cycling negatively affects bone health (low BMD). Thus, it is necessary to establish a multifactorial approach to slow down this bone deterioration. For example, high-impact jump training has been seen to be included as part of the training program for elite cyclists [62] to help stimulate bone formation with the high impact forces. However, this type of training should be performed progressively, as it may increase the risk of stress fractures, especially in individuals with BMD values of osteopenia or osteoporosis [63,64]. Therefore, an effective methodology to prevent bone deterioration would be to incorporate this type of training in youth cyclists and continue it throughout their lives [62]. In addition, establishing a nutritional plan that covers energy, protein and carbohydrate requirements and supplementation with vitamin D, Ca^+^ and collagen peptides in professional cyclists could reduce the risk of LEA and its consequences at a metabolic level, as well as positively affecting bone health [65,66,67,68,69].

The limitations of this study were the size of the sample, as access to these types of subjects is very complicated, and they move around a lot geographically, affecting their availability. In addition, factors such as diet and supplementation, bone metabolism markers and strength training were also not analyzed. This study did not include a control group.

## 5. Conclusions

One season of professional cycling (training and competition) is enough to decrease markers of bone health. This situation would increase the risk of bone fracture in the medium and long term in these athletes. Therefore, it would be necessary to establish treatments focused on preventing this bone deterioration, which could be included in the professional cyclist’s annual program.

## Figures and Tables

**Table 1:** Baseline general characteristics of the professional cyclists.

Characteristics	
Age (years)	27.3 (3.40)
Body mass (kg)	72.7 (5.98)
Height (cm)	180.0 (5.89)
FM (kg)	5.01 (1.74)
FFM (kg)	64.7 (4.88)

Values are expressed as mean (SD). FM = fat mass; FFM = fat-free mass.

**Table 2:** Comparison of bone mineral density (BMD), bone mineral content (BMC), bone area, fat mass and fat-free mass values in professional cyclists between 2018 and 2019 pre-seasons. Values are mean (SD).

	2018	2019	Time *p*-Value	ES
BMD Arms	1.11 (0.08)	1.10 (0.07)	0.102	0.407
BMD Legs	1.31 (0.10)	1.30 (0.11)	0.017 *	0.504
BMD Trunk	0.868 (0.07)	0.851 (0.07)	0.012 *	0.489
BMD Ribs	0.678 (0.04)	0.663 (0.04)	0.003 *	0.814
BMD Pelvis	1.05 (0.11)	1.02 (0.10)	0.001 *	0.811
BMD Spine	1.01 (0.10)	1.03 (0.11)	0.081	0.278
BMD Total	1.19 (0.08)	1.18 (0.08)	0.061	0.410
BMC Arms	434 (51.4)	452 (66.7)	0.033 *	0.320
BMC Legs	1213 (106)	1195 (107)	0.663	0.235
BMC Trunk	722 (125)	711 (130)	0.260	0.275
BMC Ribs	262 (49.5)	265 (62.9)	0.499	0.092
BMC Pelvis	248 (52.2)	244 (53.4)	0.427	0.192
BMC Spine	212 (36.5)	201 (38.5)	0.003 *	0.648
BMC Total	2879 (315)	2869 (322)	0.187	0.324
BA Arms	390 (34.0)	400 (30.9)	0.023 *	0.587
BA Legs	923 (44.2)	906 (134)	0.257	0.139
BA Trunk	827 (87.3)	832 (123.0)	0.459	0.063
BA Ribs	385 (53.5)	399 (87.5)	0.528	0.242
BA Pelvis	235 (30.1)	239 (40.7)	0.794	0.176
BA Spine	208 (20.2)	194 (24.1)	0.003 *	0.828
BA Total	2391 (147)	2436 (190)	0.138	0.396
FM Arm left	221 (76.0)	232 (94.7)	0.337	0.233
FM Arm right	241 (80.9)	249 (99.8)	0.446	0.184
FM Trunk	2151(741)	2008 (657)	0.459	0.291
FM Leg left	1025 (445)	997 (422)	0.865	0.120
FM Leg right	1041 (445)	1021 (442)	0.865	0.084
Fat Mass Total	5014 (1737)	4835 (1715)	0.640	0.177
FFM Arm left	3645 (417)	3719 (380)	0.097	0.414
FFM Arm right	3976 (498)	4009 (392)	0.535	0.149
FFM Trunk	27,394 (2355)	27,290 (2267)	0.638	0.113
FFM Leg left	12,463 (937)	12,527 (895)	0.470	0.174
FFM Leg right	12,696 (934)	12,811 (865)	0.159	0.348
Fat-Free Mass Total	64,722 (4883)	64,880 (4636)	0.629	0.116
T-score	−0.436 (0.965)	−0.679 (1.012)	0.061	0.462
Z-score	−0.057 (0.958)	−0.343 (1.000)	0.021 *	0.556

* = *p*-values ≤ 0.05 and trends in the range of 0.05–0.07. BA = bone area; BMC = bone mineral content; BMD = bone mineral density; FFM = fat-free mass; FM = fat mass; T-score = is the number of SD below the mean BMD in young adults, osteoporosis is defined by a BMD T-score of less than ≥2.5SD, and osteopenia by a T-score between −1 and −2.5SD.; Z-score = is the number of SDs above or below the mean BMD in the population of the same age as the patient/subject, a Z-score of −2.0SD or below was described as “low BMD for chronological age”, and above −2.0 was described as “normal BMD for chronological age”.

## Data Availability

Data supporting reported results are available upon request.
